# Adrenal gland inclusions in patients treated with aldosterone antagonists (Spironolactone/Eplerenone): incidence, morphology, and ultrastructural findings

**DOI:** 10.1186/1746-1596-9-147

**Published:** 2014-08-09

**Authors:** Kishan A Patel, Edward P Calomeni, Tibor Nadasdy, Debra L Zynger

**Affiliations:** Department of Pathology, The Ohio State University Medical Center, 410 W 10th Ave, 401 Doan Hall, Columbus, OH 43210 USA

**Keywords:** Adrenal, Spironolactone, Eplerenone, Ultrastructural, Inclusions

## Abstract

**Abstract:**

**Background:**

Spironolactone is often used to treat hypertension caused by hyperaldosteronism, and as a result, can form concentrically laminated electron dense spironolactone body inclusions within the adrenal gland. Spironolactone bodies have not been investigated in a contemporary cohort or in patients treated with the more recently approved aldosterone antagonist, eplerenone.

**Methods:**

Spironolactone bodies were retrospectively investigated in patients treated for hyperaldosteronism (n = 15) from 2012-2013 that underwent a subsequent adrenalectomy.

**Results:**

Inclusions were identified in 33% of patients treated with aldosterone antagonists, far less than previously reported. Remarkably, 50% of patients treated with spironolactone had inclusions while no patients using eplerenone alone had inclusions. Two patients treated with spironolactone had bodies present longer than the duration described in prior studies. Inclusions unexpectedly persisted in 1 patient despite increased duration of discontinued pharmacological treatment. A spectrum of histologic and ultrastructural findings were encountered within an adrenal cortical adenoma from a patient treated with both spironolactone and eplerenone. Ultrastructural examination revealed laminated electron dense bodies with the appearance of classic spironolactone inclusions as well as electron dense bodies without laminations and laminated bodies without electron dense cores.

**Conclusions:**

Our incidence rate of spironolactone bodies was much lower than previously reported, with no inclusions seen in patients treated solely with the newer aldosterone antagonist, eplerenone. Pathologists should be aware of these infrequently encountered inclusions, particularly as the clinical history of hyperaldosteronism and pharmacologic treatment may not be provided.

**Virtual Slides:**

The virtual slide(s) for this article can be found here: http://www.diagnosticpathology.diagnomx.eu/vs/4597918761268031

## Background

A common cause of primary hyperaldosteronism is an aldosterone-producing adenoma, which is initially treated with aldosterone antagonists such as spironolactone and eplerenone. As spironolactone treatment may cause limiting side effects, namely menstrual irregularities in women and impotence, decreased libido, and gynecomastia in men, eplerenone, a more recently developed drug may be used. Adrenalectomy is performed if the drugs prove ineffective. Spironolactone body formation has been well documented and occurs within the adrenal gland secondary to spironolactone treatment [1]. The composition of these bodies is purported to be a high content of Luxol fast blue staining phospholipid and aldosterone in the outer concentric laminations [2–4].

We investigated the incidence of spironolactone bodies within a recent cohort of patients treated with aldosterone antagonists, including both spironolactone and eplerenone, with a lower frequency identified than in earlier studies. Inclusion bodies were detected with extended use of spironolactone and with longer periods of discontinued use, which has not been previously reported. Additionally, we describe the spectrum of histological and ultrastructural findings in an aldosterone-producing adenoma from a patient treated with both spironolactone and eplerenone. The ultrastructural features included bodies with spiral and non-spiral membranes and bodies without membranes.

## Methods

A retrospective search was performed to identify patients treated with an adrenalectomy during a 2 year time period (January 2012-December 2013) at our hospital with a clinical history of hyperaldosteronism conducted in compliance with our Institutional Review Board (The Ohio State University Medical Center; Protocol #2002H0089) and in compliance with the Helsinki Declaration. Aldosterone antagonist treatment regimens were recorded. All slides were reviewed using light microscopy to evaluate for the presence of inclusions.

One patient with unusual inclusions was further investigated with immunohistochemical, special stains, and ultrastructural examination (patient 1, described below). Transmission electron microscopic examination was performed for this patient using formalin-fixed tissue. Formalin-fixed tissue was postfixed in glutaraldehyde (1 hour) followed by aqueous osmium tetroxide (1 hour) and uranyl acetate-lead citrate (1 hour). A second patient with classic inclusions was further investigated with ultrastructural examination using formalin-fixed paraffin-embedded tissue (patient 2, described below). A representative area of the tumor was cut from the paraffin block. The tissue was deparaffinized in 1% aqueous osmium tetroxide dissolved in toluene (4 hours). Epoxy resin infiltration was performed and the resin was polymerized to make electron microscopic blocks. Blocks were thin sectioned and examined with a JEOL JEM 1400 transmission electron microscope.

## Results

Fifteen patients were identified that had an adrenalectomy for serologically proven hyperaldosteronism. Six patients were treated with spironolactone alone, 5 were treated with eplerenone alone, 1 was treated with both spironolactone and eplerenone, and 3 did not receive pharmacologic therapy (Table 1). Inclusions were histologically identified in 4 out of the 12 treated (33.3%) and none of the untreated patients. The single patient with a history of both spironolactone and eplerenone treatment had unusual inclusions (patient 1). In the spironolactone-only subgroup, 50% had inclusions (patients 2-4). No patients using eplerenone alone had inclusions. Patient 1 discontinued spironolactone more than 4 months before surgery and was switched to eplerenone, which was discontinued for more than 21 days before surgery. Despite being off both medications for more than 3 weeks, inclusions were found. Two of 3 patients given spironolactone alone for over 100 days had bodies, and 2 of these patients treated for over 270 days had focal or diffuse bodies (patient 2 and 3).

**Table 1:** **Adrenalectomy findings in patients with hyperaldosteronism**

Case	Age	Gender	Medication (duration)	Adrenalectomy diagnosis	Spironolactone-like bodies present
1	39	M	Spironolactone (6 weeks), Eplerenone (6 weeks with no treatment 3 weeks prior to surgery)	Adrenal cortical adenoma (1.9 cm)	Diffuse
2	59	F	Spironolactone (>9 months)	Adrenal cortical adenoma (0.9 cm)	Diffuse
3	56	M	Spironolactone (>9 months)	Adrenal cortical adenoma (2.8 cm)	Focal
4	61	M	Spironolactone (>2 months)	Nodular cortical hyperplasia	Focal
5	35	M	Spironolactone (5 months)	Adrenal cortical adenoma (1.8 cm)	None
6	60	M	Spironolactone (2 months with no treatment 7 months prior to surgery)	Nodular cortical hyperplasia	None
7	68	M	Spironolactone (>2 months)	Vascular cyst	None
8	60	M	Eplerenone (>3 years)	Adrenal cortical adenoma (1.6 cm)	None
9	48	M	Eplerenone (>3 months)	Nodular cortical hyperplasia	None
10	51	M	Eplerenone (>3 years)	Nodular cortical hyperplasia	None
11	46	M	Eplerenone (>1.5 years)	Hematoma (post venus sampling)	None
12	46	M	Eplerenone (unknown)	Lipoma	None
13	43	F	No treatment	Adrenal cortical adenoma (2.3 cm)	None
14	34	F	No treatment	Adrenal cortical adenoma (1.5 cm)	None
15	62	M	No treatment	Adrenal cortical adenoma (2.3 cm)	None

### Patient 1

A 39 year-old male with known high aldosterone presented with complaint of abdominal pain. CT scan showed a 1.7 cm left adrenal lesion and a separate left lower pole kidney mass. Robotic partial nephrectomy resected a papillary type 1 renal cell carcinoma. Post-operative follow-up found continuing levels of high aldosterone and the patient was started on spironolactone (dose not documented). The patient continued to have high levels of aldosterone at 6 weeks and was switched to eplerenone 50 mg/day. Failure to lower aldosterone levels called for adrenal vein sampling, which confirmed the left adrenal nodule as the source of the hyperaldosteronism. Eplerenone dosage was increased to 50 mg twice a day after 3 weeks, then 50 mg twice in the morning and once at night after an additional 3 weeks. The patient was taken off eplerenone and underwent a unilateral adrenalectomy, resulting in the resolution of the hyperaldosteronism.

**Figure 1:**
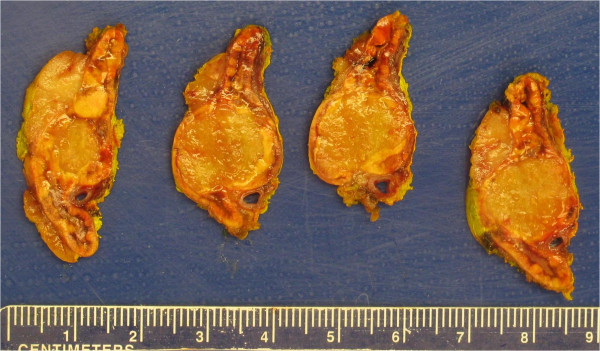
**Gross photographs of the left adrenal gland from patient 1 conteining a well-circumscribed tan-yellow cortical nodule measuring 1.9 cm.**

**Figure 2:**
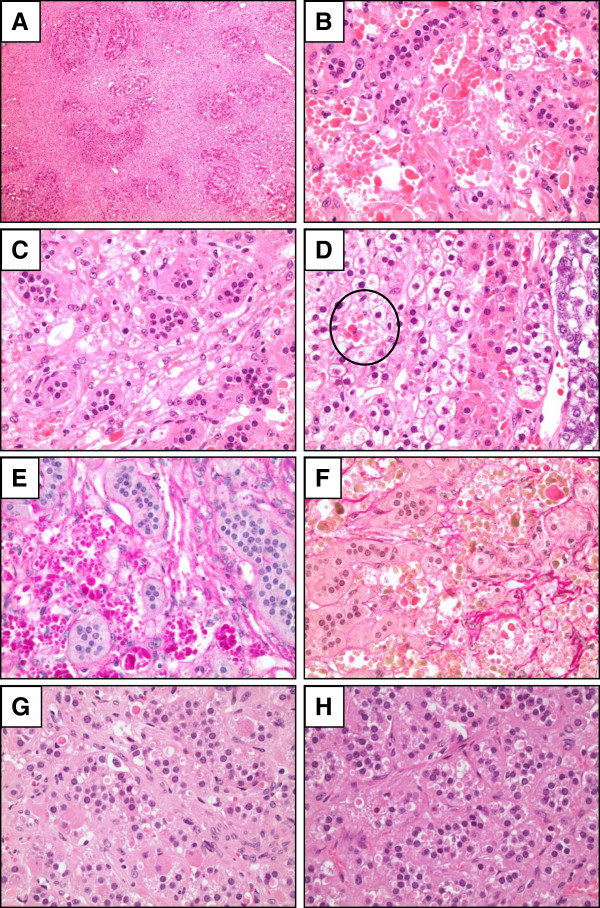
**Photomicrographs of patient 1 (A-F), patient 2 (G), and patient 4 (H). A**. Nodules of adrenal cortical tumor cells separated by an infiltrate of paler spindle cells in patient 1 (400x). **B**. Adrenal cortical tumor cells showing numerous brightly eosinophilic inclusions of varying sizes in the cytoplasm. Multiple inclusions are present in each cell (400x). **C**. Spindle cell infiltrate composed of degenerating adrenal cortical cells (400x). **D**. Inclusions were also present in the zona fasciculata directly adjacent to the tumor (400x). **E**. Inclusions positive for PAS, showing bright pink staining, with Luxol Fast Blue counterstaining. There were variably sized multiple intracellular inclusions (400x). **F**. Hemoglobin stain with a spectrum of staining including olive green, pale pink, and brick red inclusions. Multiple inclusions of each color were present in a single cell (400x). **G**. In patient 2, large eosinophilic bodies with concentric rings were found single per cell throughout the tumor (400x). **H**. In patient 4, rare large, eosinophilic, concentrically laminated bodies were seen within the zona glomerulosa (400x).

A 19.8 g, 7.5 × 3.8 × 1.8 cm left adrenal gland with attached adipose was serially sectioned to reveal a tan-yellow, rubbery cortical nodule measuring 1.9 × 1.6 × 1.5 cm (Figure 1). The mass was homogenous with no areas of hemorrhage or necrosis, and did not involve the periadrenal fat. Light microscopic examination of hematoxylin and eosin stain stained slides demonstrated a well circumscribed, unencapsulated mass composed of nests of clear to eosinophilic adrenal cortical tumor cells interspersed with degenerating bland adrenal cortical cells (Figure 2A). Numerous brightly eosinophilic inclusions of varying sizes (less than 2 μm to 50 μm) were present within the cytoplasm of the viable (Figure 2B) and degenerative (Figure 2C) adrenal cortical tumor cells. Most of the viable adrenal cortical tumor cells containing inclusions resembled zona reticularis, although rare cells with inclusions appeared similar to zona fasciculata. Inclusions varied from 0 per cell to greater than 20. In nontumoral adrenal tissue, inclusions were only present in the zona fasciculata directly adjacent to the tumor (Figure 2D).

Immunostains were performed in which the viable adrenal cortical tumor cells expressed AE1/3 (weak, diffuse), melan A (strong, diffuse), and inhibin (moderate, diffuse), but were nonreactive for PAX8, CAIX, CD68, and CD163. Degenerating cells were negative for CD68 and CD163. Special stains demonstrated that the inclusions were negative for Luxol fast blue performed with and without Periodic acid-Schiff (PAS) counterstain and negative for Prussian blue. Inclusions were positive for PAS, with bright pink staining (Figure 2E). A hemoglobin special stain was performed which variably stained the inclusions olive green, pale pink, and brick red (Figure 2F).

**Figure 3:**
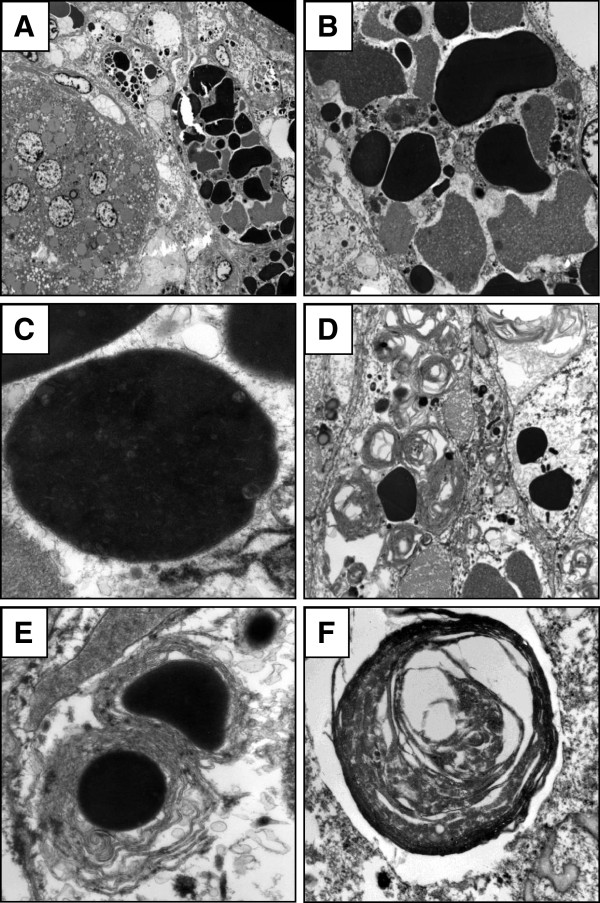
**Transmission electron micrographs of patient 1 (A-E) and patient 2 (F). A**. Classic adenoma cells on the left. The cells on the right contained dense inclusions (2,000x). **B**. Some of the cells had multiple inclusions with variable electron density (6,000x). **C**. This election dense inclusion may represent a degenerated mitochondrion containing possible remnant cristae as it is surrounded by a membrane. However, most of the dense inclusions had no lining membrane (60,000x). **D**. Inclusions with characteristic concentrically laminated membranes similar to those surrounding typical spironolactone bodies, but without an electron dense core (12,000x). **E**. Some cells contained inclusions similar to typical spironolactone bodies, complete with an electron dense core surrounded by concentrically laminated membranes (40,000x). **F**. In patient 2, using paraffin-embedded tissue, only scroll-like inclusions without electron dense cores were identified (60,000x).

Ultrastructural examination using formalin-fixed tissue revealed that most classic viable adenoma cells did not contain dense inclusions. These cells had numerous organelles, including slightly swollen mitochondria and weakly electron dense lipid droplets (Figure 3A). Some viable and degenerative adrenal cortical cells contained inclusions. The size of the bodies varied and multiple morphologies were seen in a single cell. There were some inclusions touching the nucleus while others were in proximity to mitochondrion or endoplasmic reticulum. The inclusions had variable electron density, which may be due to the varying amount of unsaturated lipids and/or degenerative change (Figure 3B). A few inclusions looked like they may have originated from degenerated mitochondria due to possible remnant cristae and the presence of a membrane (Figure 3C). However, most of the dense inclusions did not have such structure. Lipofuscin granules were seen admixed with the dense inclusions. There were several inclusions that resembled typical spironolactone bodies with a scroll-like membrane but without an electron dense core present (Figure 3D). There were only a few bodies with both a scroll-like concentric membrane and an electron dense core (Figure 3E).

### Patient 2-4

Inclusions were histologically identified in an additional three patients. A 59 year-old female with a history of hypertension since age 25 was started on spironolactone 25 mg once a day for more than 9 months (patient 2). The adrenalectomy contained a 0.9 cm adrenal cortical adenoma. Large pale eosinophilic bodies with concentric rings were found single per cell throughout the tumor, with minimal variation in size (Figure 2G). Ultrastructural examination was performed using paraffin-embedded tissue as no other tissue was available. Only inclusions with scroll-like membranes without electron dense cores were identified (Figure 3F). A 56 year-old male with a history of poorly controlled hypertension and obesity was started on spironolactone 100 mg twice a day for more than 9 months (patient 3). The adrenalectomy resected a 2.8 cm adrenal cortical adenoma. Rare bodies were seen, appearing as large concentrically laminated inclusions, single per cell, only in focal areas of the tumor. A 61 year-old male with a history of hypertension for 15 years was started on spironolactone 50 mg once a day for more than 2 months (patient 4). Nodular cortical hyperplasia was diagnosed in the adrenalectomy. Histology revealed rare eosinophilic, large, and concentrically laminated bodies within the zona glomerulosa (Figure 2H).

## Discussion

We investigated spironolactone bodies of the adrenal gland within a recent 2 year period in patients with hyperaldosteronism. Spironolactone bodies occur in the adrenal gland secondary to spironolactone treatment [1] but have yet to be investigated in patients treated with eplerenone, a newer aldosterone antagonist.

The incidence of spironolactone bodies within the adrenal gland in contemporary patients taking spironolactone or eplerenone is unknown. In studies published between 1981 and 1985, inclusions were found within 74-100% of patients with primary hyperaldosteronism that were pharmacologically treated with spironolactone [3, 5–7]. We detected inclusions in only 33% (4/12) of patients with primary hyperaldosteronism treated with spironolactone and/or eplerenone, much lower than prior studies. Remarkably, 50% of patients treated with spironolactone had inclusions while no patients using eplerenone alone had inclusions. It is possible that the release of eplerenone in 2002 and its subsequent preferred use due to less undesirable side effects have decreased the incidence of spironolactone-like bodies.

The spironolactone bodies in some of our patients persisted much longer than the duration for which spironolactone bodies are thought to occur. Spironolactone bodies decrease over time as spironolactone continues to be administered [6, 8]. Conn et al. found the number of spironolactone bodies peaked 4-6 weeks after the start of treatment after which there was a marked decrease, with a third of patients treated for 100-170 days having no detectable bodies [8]. These authors suggested that the existence of bodies gradually diminishes to zero by 170 days [8]. However, in our study, 67% of patients treated with spironolactone alone for over 100 days had bodies and 2 patients treated for over 270 days were found to have either diffuse or focal bodies. The effect of dosage on the persistence of bodies is unknown, but it is of note that in the report by Conn et al., patients with no inclusions received higher dosages of spironolactone [8]. Spironolactone bodies eventually disappear if medication is discontinued [6, 8]. Authors have shown that discontinuation of spironolactone 18-97 days before surgery results in no identifiable inclusion bodies [6, 8]. However, patient 1 in our study discontinued medications for more than 3 weeks and yet inclusions were found. Eplerenone administration may have resulted in these bodies occurring longer than expected.

The ultrastructural morphology of typical spironolactone bodies show scroll-like concentric laminations of membranes with an electron-dense core [1, 2, 5]. Inclusion bodies with unusual histologic features were identified in 1 of our patients and were subsequently examined ultrastructurally, showing a spectrum of findings. A few bodies resembling typical spironolactone bodies were present but there were also inclusions that had the same scroll-like appearance but did not have a visible electron dense core typical for fully-developed spironolactone bodies. Additionally identified were dense inclusions that did not have the concentric laminations or any membrane typical of spironolactone bodies. The specificity of spironolactone bodies for spironolactone treatment is under question. Several researchers have stated that these bodies appear exclusively in aldosterone-producing cells [8, 9]. Kovacs et. al concluded that spironolactone bodies are not exclusively due to spironolactone treatment based on the presence of similar “fingerprint-like bodies” elsewhere in the adrenal cortex secondary to treatment with other drugs [2].

Spironolactone bodies have been histologically depicted in the literature as typically solitary eosinophilic bodies surrounded by a clearing within a single cell [10]. However, 1 of our specimens histologically contained numerous inclusions which ultrastructurally were composed of both typical spironolactone bodies and membrane-less electron-dense inclusions in individual cells. Mete and Asa described similar membrane-less electron-dense inclusions in a patient with an adrenal cortical adenoma [11]. Unlike our case, their patient was not treated with any aldosterone antagonist and did not have any typical spironolactone bodies in that the inclusions were not concentric or laminated [11]. The bodies that these authors found were multiple per cell and histologically appeared similar to those we identified. Of note, formalin-fixed paraffin embedded tissue was used. Formalin-fixed paraffin embedded tissue has been found by previous studies to be suboptimal for ultrastructural examination and in 1 of our patients only revealed scroll-like inclusions that lacked electron dense cores [12, 13].

The location in which spironolactone bodies are found is variable. Of our 3 patients that had an adrenal cortical adenoma with inclusions bodies, 2 had inclusions only in the tumor, while 1 had bodies predominately in the tumor but also in the non-neoplastic adrenal tissue adjacent to the tumor, mostly in the zona fasciculata and rarely in the zona glomerulosa. In 1 patient with nodular cortical hyperplasia and bodies, the inclusions were confined to the zona glomerulosa. Similar to our results, most authors have identified bodies in the tumor only, while others have found bodies in the tumor and adjacent tissue [3, 6, 11].

Typical spironolactone bodies are described to be Luxol fast blue positive and PAS negative [2, 3]. They are hypothesized to originate from the smooth endoplasmic reticulum [2, 3, 14]. The inclusion is thought to be rich in phospholipids due to Luxol fast blue staining [2, 3]. The concentric laminations have been demonstrated to contain aldosterone [4]. However, we found the bodies to be negative for Luxol fast blue and positive for PAS. Mete and Asa also reported similar Luxol fast blue negativity and PAS positivity [11]. Future studies are needed to further investigate the contents of these inclusions using more sophisticated techniques.

## Conclusion

In conclusion, we found the incidence rate of spironolactone bodies to be far less than reported in the past, with no inclusions identified in cases in which only eplerenone was used. Bodies were present after their typical life-span and were seen after medication was discontinued for an extended period of time. Additionally, we describe histologically unusual inclusions with both classic and more uncommon ultrastructural findings of spironolactone bodies. Surgical pathologists should be aware of these infrequently encountered inclusions to prevent misinterpretation and unnecessary work-up.
